# Halloysite Nanotubes as Dynamic Interfaces for Functional Enzyme Biomaterials

**DOI:** 10.1021/acsomega.6c06020

**Published:** 2026-08-17

**Authors:** Giuseppe Cinà, Marina Massaro, Federica Leone, Claudia Sciacca, Nunzio Cardullo, Vera Muccilli, Angelo Nicosia, Placido G. Mineo, Rita Sánchez-Espejo, César Viseras, Serena Riela

**Affiliations:** † Dipartimento di Scienze Chimiche (DSC), Università di Catania, Viale Andrea Doria 6, 95125, Catania, Italy; ‡ Dipartimento di Scienze e Tecnologie Biologiche, Chimiche e Farmaceutiche (STEBICEF), Università degli Studi di Palermo, Viale delle Scienze, Parco d’Orleans II, Ed. 17, 90128 Palermo, Italy; § Department of Pharmacy and Pharmaceutical Technology, School of Pharmacy, Campus of Cartuja s/n, 16741 University of Granada, 18071, Granada, Spain; ∥ Andalusian Institute of Earth Sciences, CSIC-UGR, 18100 Armilla, Granada, Spain

## Abstract

The development of robust and recyclable enzyme-based materials is essential for advancing sustainable biocatalysis and biotechnologies. Herein, we report the design of covalent enzyme-halloysite biohybrid materials obtained by the controlled functionalization of halloysite nanotubes (HNTs), a naturally abundant, low-cost, and biodegradable clay nanomaterial acting as a dynamic biological interface. Two structurally and functionally distinct enzymes, collagenase G (ColG) and laccase (Lac), were selected as model systems to investigate how the halloysite surface can modulate the enzyme organization and catalytic behavior. Through tailored interfacial functionalization of the HNT external surface, stable enzyme-clay biointerfaces were obtained while preserving enzymatic activity. Comprehensive spectroscopic, thermal, and microscopic analyses confirmed the successful formation of homogeneous hybrid biointerfaces and revealed distinct supramolecular organization, depending on the immobilized enzyme. Enzymatic assays demonstrate that both HNTs-ColG and HNTs-Lac retained their catalytic function after surface conjugation. Notably, the laccase-based biomaterial efficiently degrades methyl orange under mediator-free conditions, a behavior rarely observed in halloysite-supported laccase systems, while operating under both batch and continuous flow conditions and exhibiting high recyclability over multiple cycles. These findings suggest that halloysite nanotubes do not merely behave as a passive support but rather as adaptive inorganic-biological interfaces capable of dynamically influencing enzyme function through interfacial effects, in particular, via the covalent-induced conformational modulation of the enzyme after its grafting onto the external HNTs surface. The proposed strategy opens new perspectives for the development of functional enzyme materials for bioremediation and biomedical applications.

## Graphical abstract

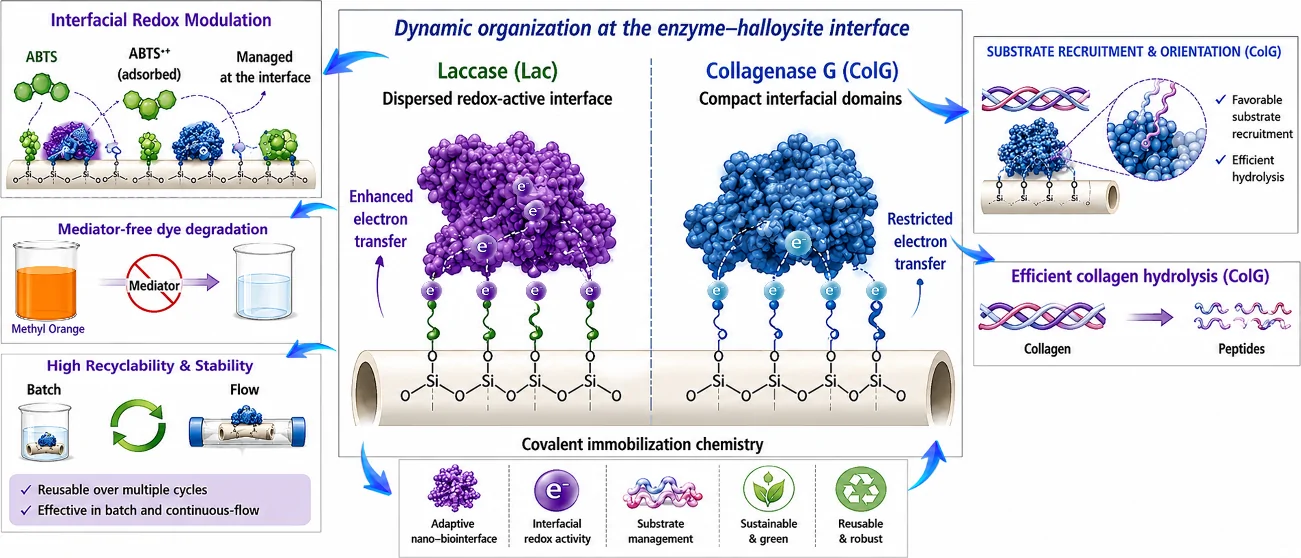

## 1 Introduction

The development of efficient and recyclable biocatalytic systems is a central objective in sustainable chemistry and biotechnology.^1^ Enzymes play a pivotal role in this transition, owing to their ability to perform highly selective transformations under mild operating conditions while minimizing energy consumption and hazardous byproducts.^2,3^ Nevertheless, their practical implementation is still limited by structural fragility, sensitivity to environmental conditions, and poor operational stability, particularly in heterogeneous and continuous processes.^4,5^ Conventional immobilization approaches have been extensively investigated to enhance enzyme durability, storage stability, and reusability.^6^ Over time, several immobilization methods have been proposed, including physical adsorption, which relies on hydrogen-bonding, van der Waals forces, and ionic interactions.^6–8^ Various materials have been explored as immobilization supports, such as zeolites, natural and synthetic polymers, and nano- and microparticles.^9,10^ However, emerging perspectives in biohybrid materials suggest that the inorganic scaffold should not merely act as a passive support for enzyme anchoring. Instead, increasing attention is being devoted to the design of dynamic inorganic-biological interfaces capable of influencing enzyme organization, interfacial accessibility, substrate diffusion, and catalytic behavior.^6,11^ In this context, clay minerals, and particularly halloysite, in its nanotubular form (HNTs), have recently emerged as promising building blocks for advanced biohybrid materials and biotechnological systems.^12–16^ HNTs are abundant, low-cost, nontoxic, and biodegradable aluminosilicates, characteristics that make them highly attractive within the framework of sustainable chemistry and circular economy. Beyond their intrinsic sustainability, HNTs possess a unique nanotubular morphology and chemically differentiated surfaces, with siloxane groups predominantly exposed on the external surface, some silanol groups as structural defects, and aluminol groups lining the inner lumen. This dual-surface chemistry enables selective interfacial engineering and controlled organization of organic and biological components at the nanoscale, including enzymes.^17,18^ A few reports in the literature have addressed the supramolecular immobilization of enzymes on HNT surfaces via electrostatic interactions, demonstrating the feasibility and advantages that the clay can confer to the final nanomaterial, such as enhanced stability of the enzymatic species.^17–19^ Depending on the modification approach used, rather than acting merely as passive immobilization supports, halloysite nanotubes can provide confined and adaptive microenvironments capable of influencing enzyme distribution, supramolecular organization, substrate accessibility, and interfacial catalytic behavior. Nevertheless, despite the growing interest in enzyme-halloysite systems, the potential role of HNTs as dynamic interfaces remains largely unexplored. Most reported enzyme/halloysite systems are based on reversible adsorption processes, where the mineral component mainly acts as a passive support.^20–22^ Moving beyond conventional adsorption approaches requires the development of structurally defined enzyme-halloysite interfaces in which the organization and accessibility of the biological component can be controlled at the nanoscale. Achieving this level of interfacial control requires selective chemical strategies capable of preserving enzyme structure while promoting robust communication between the biological and mineral components, an approach lying at the intersection of organic chemistry, materials science, and biotechnology.^23,24^ Within this framework, two structurally and functionally distinct enzymes, namely, laccase (Lac) and collagenase G (ColG), were selected as complementary model systems to investigate the adaptive behavior of halloysite-based biointerfaces. Collagenase G represents a relevant model for proteolytic and biomedical biocatalysis because of its ability to modulate extracellular matrix components, thus relevant for drug delivery, tissue engineering, and tumor-targeting strategies.^25,26^ Conversely, Laccase is one of the most representative enzymes in green redox biocatalysis, capable of oxidizing dyes, phenols, and persistent pollutants using molecular oxygen while producing only water as a byproduct.^27–34^ The obtained biomaterials, HNTs-Lac and HNTs-ColG, were thoroughly investigated through complementary physicochemical and morphological analyses in order to elucidate the formation and organization of the enzyme-halloysite interfaces. The enzymatic activity of both biomaterials was then evaluated to assess how the halloysite surface influenced enzyme functionality after interfacial conjugation. In particular, HNTs-Lac exhibited efficient methyl orange degradation under both batch and continuous-flow conditions without requiring any redox mediator, suggesting the emergence of interfacial effects capable of modulating the catalytic response of the immobilized enzyme as a consequence of its covalently induced conformational modulation following the grafting onto the external HNTs surface. Overall, this study highlights the potential of halloysite nanotubes as dynamic biointerfaces for the development of functional enzyme-based materials.

## 2 Results and Discussion

To investigate the ability of halloysite nanotubes to act as adaptive biointerfaces for structurally distinct enzymes, collagenase G (ColG) or laccase (Lac) was anchored onto the external surface of previously modified HNTs^35^ through a tailored covalent immobilization strategy, affording the final biomaterials HNTs-ColG and HNTs-Lac, respectively.

In particular, ColG contains several accessible carboxy-terminal amino acid residues^36^ that can be selectively functionalized without affecting enzymatic activity.^37,38^ For this reason, ColG was covalently immobilized onto amino-modified halloysite (HNTs-NH_2_)^39^ through an EDC-mediated amide coupling reaction, affording the HNTs-ColG biomaterial (Scheme 1). Thermogravimetric analysis (TGA) estimated an enzyme loading of 9.2 wt %, in comparison to the HNTs-NH_2_ precursors, consistent with the high availability of reactive residues. On the contrary, Laccase exposes fewer accessible carboxyl groups, as many reactive residues are buried within the folded protein structure rather than being solvent-accessible. Therefore, in order to improve enzyme accessibility and interfacial organization on the clay surface, the organic linker on HNTs was further modified by attaching tartaric acid to HNTs-NH_2_, producing the HNTs-COOH nanomaterial designed to ensure sufficient enzyme mobility.^35,40^ This additional functional layer was expected to provide increased flexibility at the enzyme-surface interface. HNTs-COOH was then used for Laccase immobilization via an EDC-mediated amide condensation reaction, affording the HNTs-Lac biomaterial with an enzyme loading of 1.6 wt % (Scheme 1), as estimated by TGA. The successful surface functionalization of HNTs and the integration of the enzyme component were investigated through complementary physicochemical analyses. The FT-IR spectra of HNTs-Lac and HNTs-ColG biomaterials (Figure 1a) exhibit the typical vibration bands of halloysite,^41^ along with additional bands attributed to the presence of enzymes. Specifically, within the 1650–1420 cm^–1^ range, bands corresponding to amide I (1648 cm^–1^), amide II (1563 cm^–1^), and C–N stretching (1437 cm^–1^) are clearly observed. Additionally, a band at 3100 cm^–1^, associated with -NH stretching vibrations, is present, consistent with the presence of ColG and Lac enzymes.^21,42^ Figures 1b-c show the TG curves of the HNTs-ColG and HNTs-Lac biomaterials, along with those of their respective precursors for comparison. Besides the characteristic mass loss of halloysite associated with the removal of interlayer water molecules (around 550 °C), the TG curves of both nanomaterials display additional mass losses attributed to the enzyme component. These processes are evidenced by distinct peaks centered at approximately 290 °C for HNTs-Lac and 330 °C for HNTs-ColG in the differential thermogravimetric (DTG) curves, further confirming the presence of the organic moieties on the obtained nanomaterials.

**Scheme 1:**
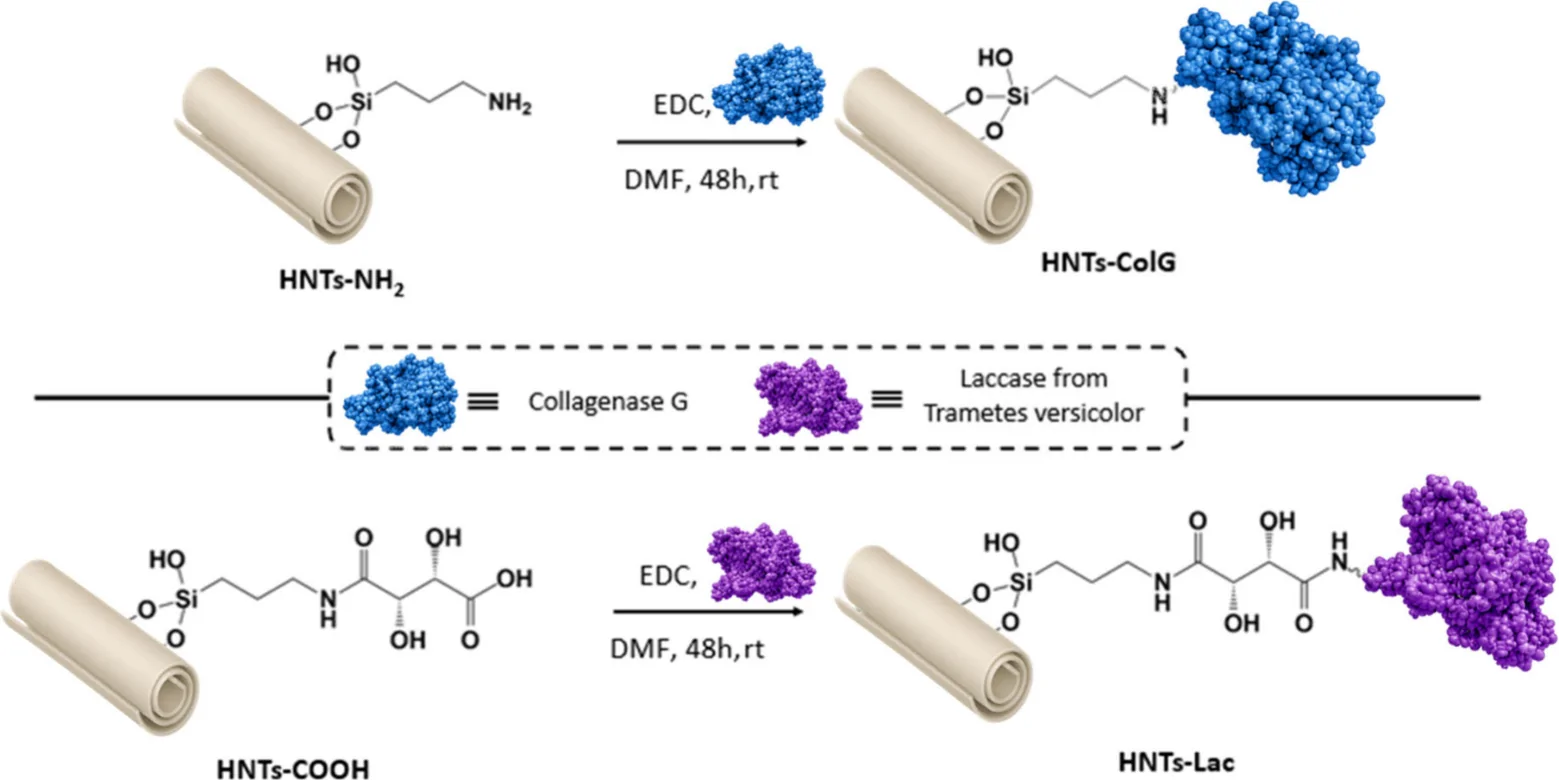
Schematic Representation of the Synthesis of HNTs-ColG and HNTs-Lac Biomaterials

**Figure 1:**
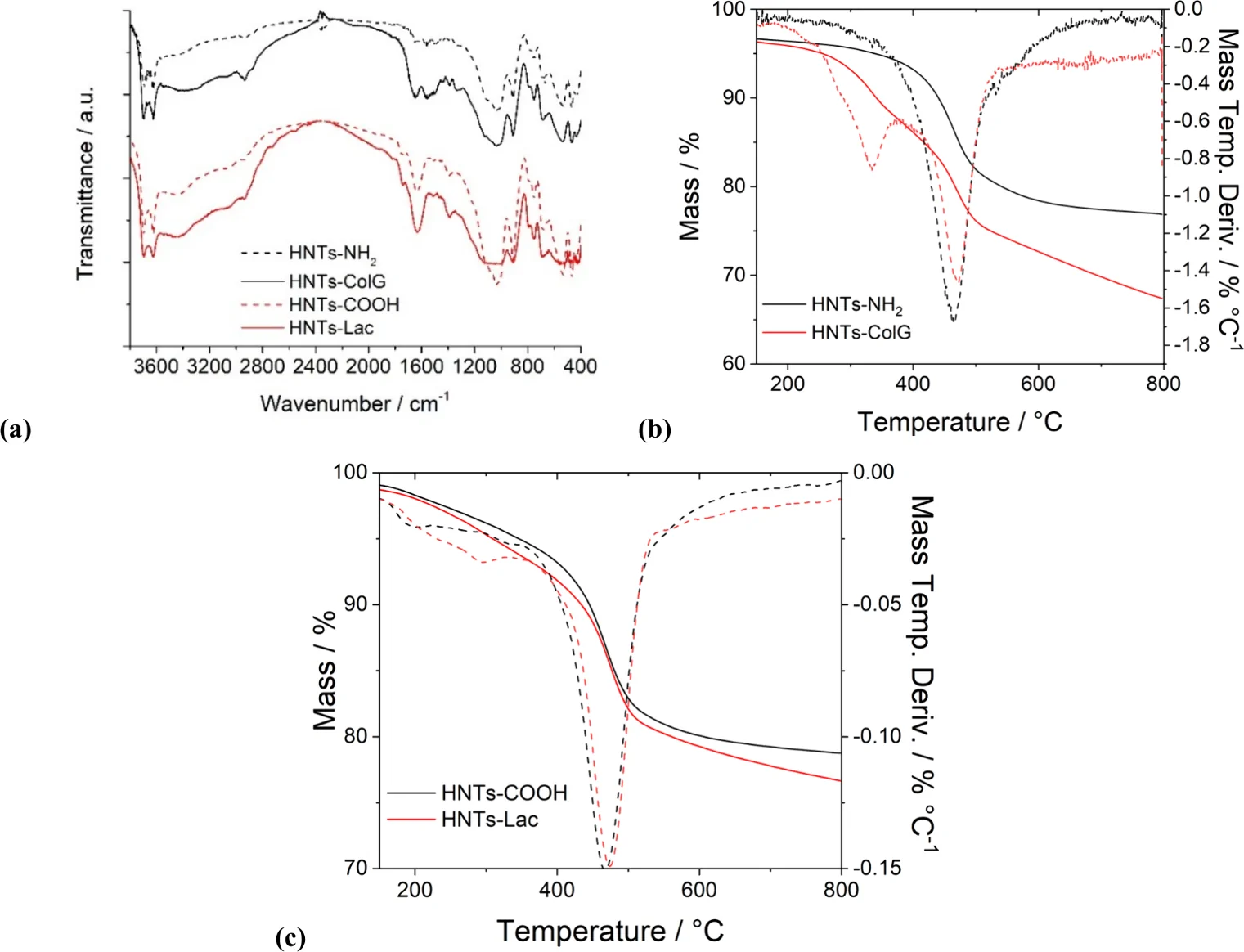
(a) FT-IR spectra of HNTs-NH_2_, HNTs-COOH, HNTs-ColG, and HNTs-Lac biomaterials; thermogravimetric curves of (b) HNTs-ColG and (c) HNTs-Lac biomaterials.

From these data, it was also possible to calculate the degree of functionalization of halloysite with both enzymes, by using the rule of the mixture^43,44^ by considering the mass losses at 25–150 °C (ML_150_) and the residual masses at 800 °C (MR_800_) for the pristine components (HNTs-NH_2_ and enzymes) and the developed nanomaterials (see SI). Dynamic Light Scattering (DLS) measurements are commonly employed to evaluate the structural characteristics of halloysite-based biomaterials. By monitoring their mobility in aqueous solutions to determine their average translational diffusion coefficients, it is possible to calculate the Z-average hydrodynamic size of the studied particles. Both HNTs-ColG and HNTs-Lac biomaterials showed, in comparison to their precursors, Z-average size values greater than 1000 nm and PDI value of 1 (Table 1) in agreement with the external surface modification, which introduced organic moieties that can interact with each other slowing the diffusion of the biomaterials in aqueous media. In comparison to their precursors, the introduction of both enzymes onto the HNTs surface increases the ζ-potential values of biomaterials (Table 1). The differences in the ζ-potential values of HNTs-Lac and HNTs-ColG biomaterials could be ascribed to the different location of carboxy-terminal amino acid residues in the enzyme pocket, as discussed above.^19,45^

**Table 1:** Z-Average and ζ-Potential Values of the Synthesized Biomaterials^a^

Sample	Z-Average size (nm)	PDI	ζ-Potential (mV)
HNTs-NH_2_	480 ± 89	0.315	–1.6
HNTs-COOH	630 ± 42	0.536	–26.0
HNTs-Lac	2553 ± 615	1.0	6.8
HNTs-ColG	1060 ± 50	1.0	–0.9

a Data are mean values ± SD of three independent assays.

SEM images revealed distinct morphological organization for two biomaterials (Figure 2a-b). In both systems, the typical tubular morphology of halloysite was clearly preserved after the enzyme conjugation. However, HNTs-ColG biomaterials exhibited a markedly more compact and interconnected architecture, where the nanotubes appeared to be coated by a smoother organic layer and organized into densely packed assemblies. Such differences likely arise from the distinct structural organization of the immobilized enzymes and from cooperative intermolecular interactions occurring at the enzyme-halloysite interface, in agreement with recent studies highlighting the dynamic role of nanobiointerfaces in modulating biomolecular organization and catalytic behavior.^46,47^ Similar evidence emerged from high-angle annular dark-field scanning transmission electron microscopy (HAADF-STEM) measurements. As it is possible to observe in Figure 2c, HNTs-ColG displayed a compact supramolecular organization compared with the more dispersed morphology observed for HNTs-Lac (Figure 2f). Such differences likely arise from the distinct structural organization of the immobilized enzymes and from cooperative intermolecular interactions occurring at the enzyme-halloysite interface. In particular, the high local density of collagenase units on the external surface of HNTs may promote a compact and organized interfacial arrangement while preserving substrate accessibility and catalytic activity, consistent with the dynamic organizational behavior often observed at nanobiointerfaces.^46,48^ Elemental mapping, extrapolated from EDX measurements, showed that N atoms, related to enzymes, were homogeneously distributed over both biomaterial surfaces (Figure 2d), confirming the uniform integration of the enzymes throughout the hybrid systems. As further proof, EDX spectra revealed the presence of N and C atoms, related to the organic portion in addition to Si, Al, and O atoms characteristic of the halloysite framework (Figure 2f).

**Figure 2:**
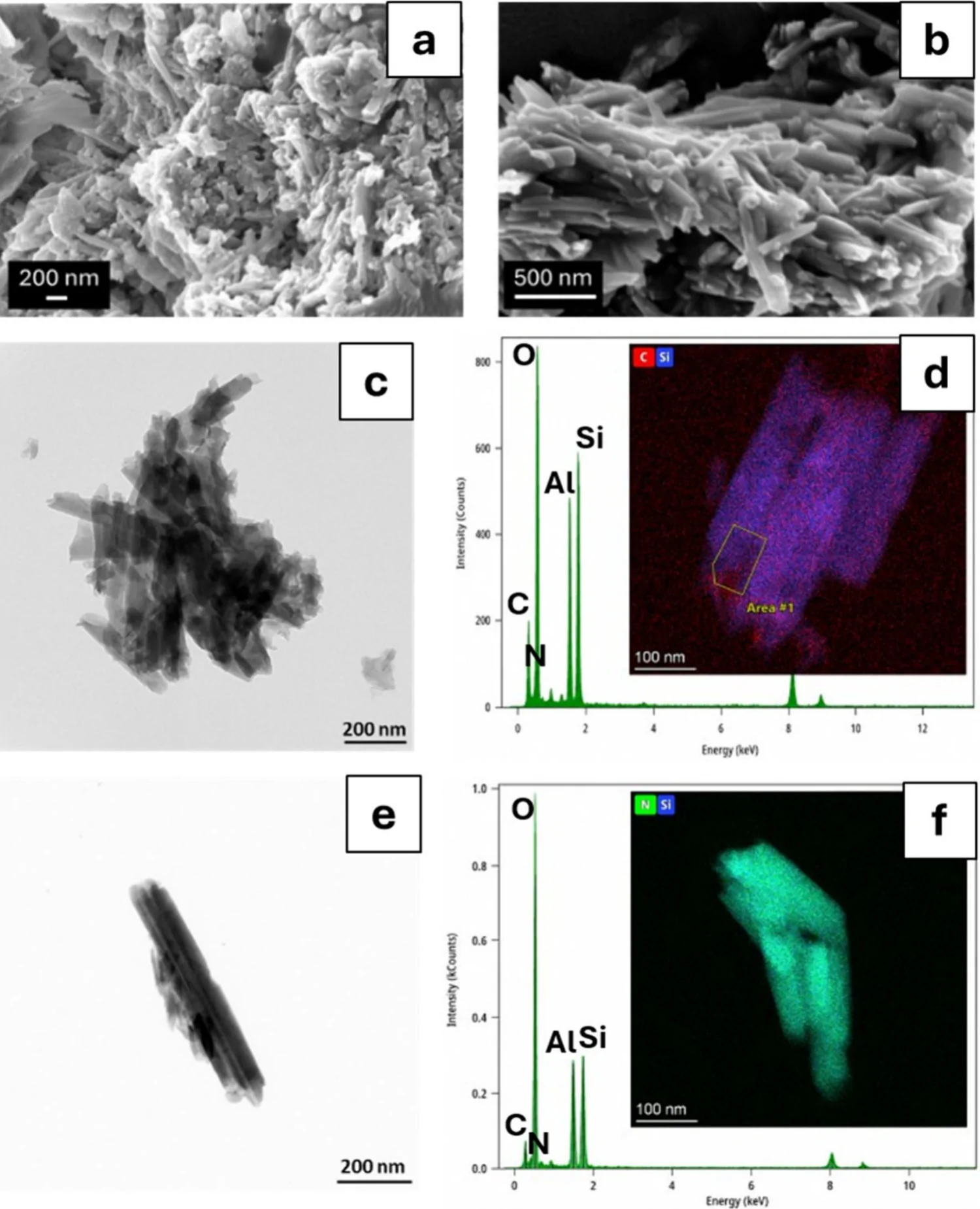
(a-b) SEM images of (a) HNTs-ColG and (b) HNTs-Lac biomaterials. (c) HAADF-STEM images, (d) EDX elemental mapping, and EDX analysis of HNTs-ColG biomaterial. (e) HAADF-STEM images, (f) EDX elemental mapping, and EDX analysis of HNTs-Lac biomaterial.

### 2.1 Enzymatic Activity Assay of HNTs-ColG and HNTs-Lac Biomaterials

The catalytic behavior of both hybrid biomaterials was investigated in order to assess how the halloysite interface influences enzyme functionality after surface conjugation.^46^ Two complementary activity assays were employed according to the specific catalytic role of each enzyme. Collagenase activity was evaluated through a fluorogenic assay monitoring collagen digestion, whereas laccase activity was investigated by following the oxidation of 2,2′-azinobis­(3-ethylbenzothiazoline-6-sulfonic acid) (ABTS).

The enzymatic activity of HNTs-ColG was evaluated by monitoring collagen degradation in the presence of 3,4-dihydroxyphenylacetic acid (3,4-DHPAA) as a fluorogenic probe. This assay was selected because of its high sensitivity toward N-terminal Gly-containing peptides (NGPs), which are generated during the collagen enzymatic digestion. In fact, 3,4-DHPAA has been shown to exhibit considerably higher sensitivity than traditional spectrofluorometric, immunological, and colorimetric techniques for detecting collagen degradation products.^49^

In brief, bovine collagen-I was incubated with a HNTs-ColG dispersion in Tris buffer at pH 7.5, in the presence of CaCl_2_ and borate solution and left to react for 60 min at 37 °C. After this time, a solution of 3,4-DHPAA and NaIO_4_ was added, and the fluorescence emission of the resulting mixture was recorded. Figure 3a shows the emission spectrum of the fluorescent NGPs-3,4-DHPAA compound, with the typical emission band at 465 nm (λ_ex_ = 365 nm), indicating the formation of collagen degradation products after enzymatic digestion of collagen-I by means of HNTs-ColG biomaterial. A comparable behavior was observed with free collagenase G (Figure S1). These findings demonstrate that the halloysite interface preserves the collagenolytic functionality of ColG after surface conjugation. Moreover, the compact supramolecular organization observed for HNTs-ColG suggests the formation of organized enzyme-rich interfacial domains capable of maintaining substrate accessibility and catalytic activity. Such features may be particularly attractive for the future development of functional biomaterials for biomedical and anticancer-related applications.^25,26^

**Figure 3:**
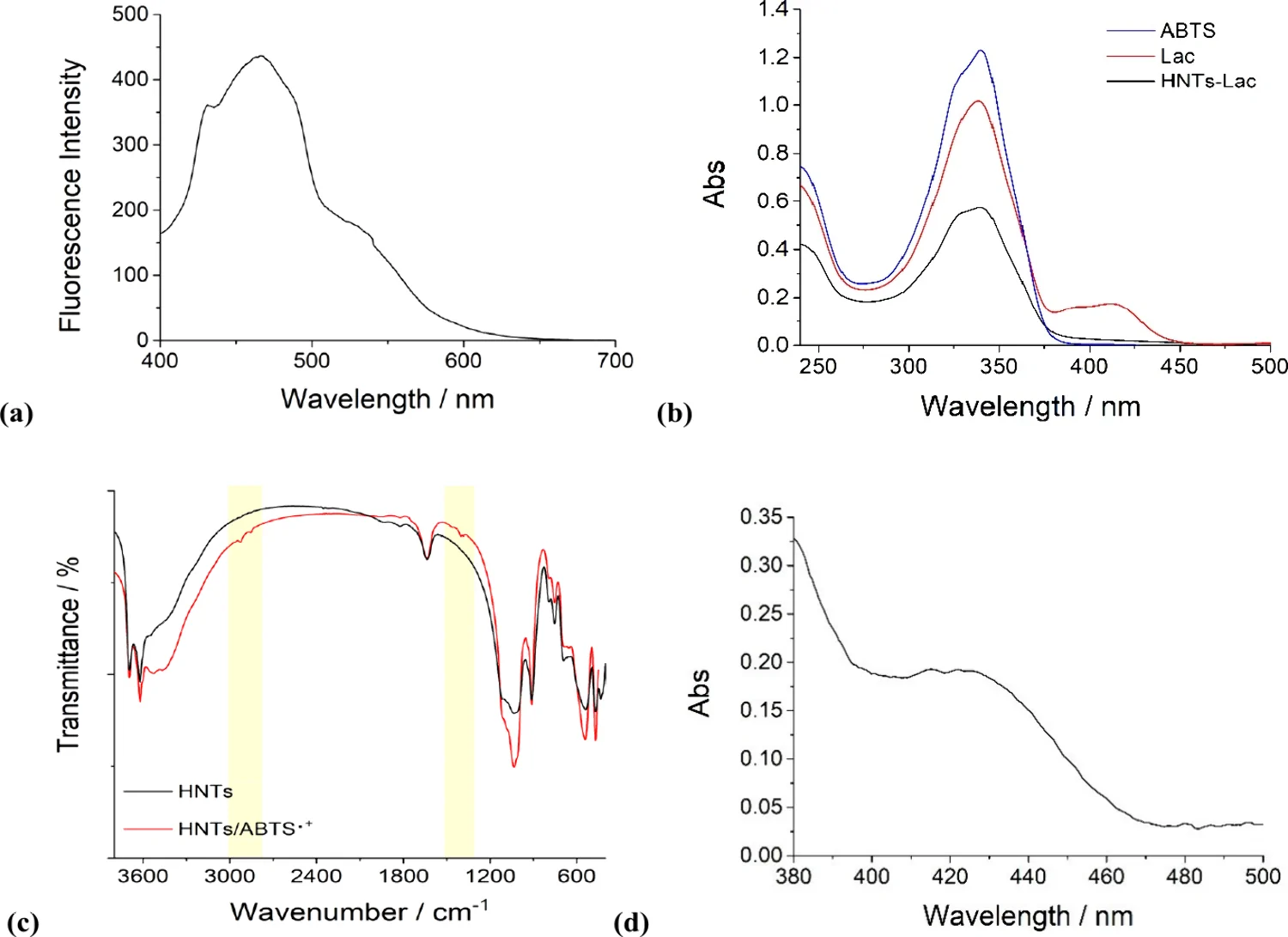
(a) Emission spectra of the fluorescent NGPs-3,4-DHPAA compound after enzymatic digestion of collagen-I by means of HNTs-ColG biomaterial (λ_ex_ = 365 nm). (b) ABTS assay (0.1 mM) of Laccase and HNTs-Lac biomaterials after 30 min of incubation. (c) FT-IR spectra of HNTs and HNTs/ABTS^•+^ biomaterials. (d) Optical fiber absorption spectrum recorded of HNTs/ABTS^•+^ biomaterial.

#### 2.1.1 Enzymatic Activity Assay of HNTs-Lac

The enzymatic activity of the HNTs-Lac biomaterial was investigated through the oxidation of ABTS, one of the most widely employed substrates for evaluating laccase catalytic activity. Upon oxidation by laccase, ABTS is converted into the radical cation ABTS^•+^ leading to the decrease of its characteristic absorption band at 340 nm together with the appearance of a new absorption band at 420 nm (Figure 3b). Pristine HNTs did not induce any oxidation toward ABTS (Figure S2). Conversely, in the presence of HNTs-Lac, a progressive decrease of the ABTS absorption band was clearly observed, indicating that the oxidation process still occurred after enzyme conjugation onto the halloysite surface. Interestingly, however, the characteristic absorption band of ABTS^•+^ at 420 nm was not initially detected. Since pristine HNTs do not alter ABTS stability in solution, it was hypothesized that electrostatic interactions occurring between the negatively charged halloysite external surface and the positively charged ABTS^•+^ radical cation promote the adsorption of it onto the clay surface immediately after its formation.^47^ In addition, the siloxane-rich external surface of halloysite may provide a relatively less hydrated and more organophilic interfacial environment capable of favoring the localization of aromatic oxidized species, consistent with the dynamic physicochemical interactions commonly governing nanobiointerfaces.^46^ To verify these hypotheses, FT-IR and solid-state UV-*vis* spectra were recorded on the solid hybrid material obtained after mixing preformed ABTS^•+^ aqueous solutions with pristine HNTs (Figures 3c-d). The FT-IR spectrum of the resulting HNTs/ABTS^•+^ complex (Figure 3c) exhibited, in addition to the characteristic halloysite bands, additional vibrational features associated with the organic radical cation. In particular, stretching vibrations of methylene groups were observed at approximately 2930–2850 cm^–1^ together with aromatic ring vibrations in the 1600–1500 cm^–1^ region. Consistently, the solid-state UV-*vis* spectrum (Figure 3d) displayed the characteristic absorption band of ABTS^•+^, clearly confirming its adsorption onto the halloysite surface. Thus, to further prove this hypothesis, the initial ABTS concentration was increased, following a procedure reported for supramolecular enzyme-clay systems.^17^ In this case, saturation of the halloysite adsorption sites was achieved and the characteristic absorption band of ABTS^•+^ at approximately 420 nm progressively appeared in solution. This observation further confirmed that the HNTs-Lac biomaterial efficiently catalyzes ABTS oxidation and simultaneously highlighted the dynamic role of the halloysite interface in modulating the distribution and interfacial fate of the generated radical species. These findings suggest that the HNTs-Lac system behaves as a dynamic redox-active biointerface in which the clay surface not only supports the enzyme organization but also participates in the interfacial management of reactive species, similarly to what has been recently proposed for adaptive nanobiointerfacial systems involved in enzymatic redox catalysis.^46,47^

### 2.2 Organic Dyes Degradation

To explore a potential environmental application of the developed biomaterials, the catalytic performance of HNTs-Lac toward wastewater remediation was investigated by monitoring the decolorization of methyl orange (MO) in an aqueous solution.

Under these conditions, HNTs could significantly attract the negatively charged MO into the positively charged lumen through both electrostatic and hydrophobic interactions, resulting in its enrichment on the biomaterial surface.

In Figure 4a, the UV–vis spectra of MO in the presence of HNTs, Lac, and HNTs-Lac (with or without ABTS as mediator) are reported. As previously reported,^49^ only negligible adsorption of MO occurs on pristine halloysite under the adopted experimental conditions. Therefore, the decrease of the characteristic absorption band of MO observed in the presence of the laccase-containing biomaterials can be reasonably attributed to catalytic dye degradation rather than to physical adsorption phenomena. Surprisingly, the HNTs-Lac biomaterial promoted approximately 70% MO degradation even in the absence of ABTS, exhibiting catalytic performances comparable to those observed in the presence of the mediator. This behavior is particularly remarkable because most reported laccase-based halloysite systems require the presence of external redox mediators to efficiently oxidize recalcitrant organic pollutants. For instance, magnetically functionalized halloysite-supported laccase systems previously reported in the literature showed effective degradation of sulfamethoxazole only under mediator-assisted conditions.^22^ The enhanced mediator-free catalytic activity observed for HNTs-Lac suggests that the halloysite interface promotes favorable interfacial conditions for dye oxidation, probably through the modulation of substrate accessibility at the enzyme-surface interface. Such findings further support the role of HNTs-Lac as a dynamic redox-active nanobiomaterial capable of extending the catalytic response of the immobilized enzyme beyond that observed for the free counterpart.^47^

**Figure 4:**
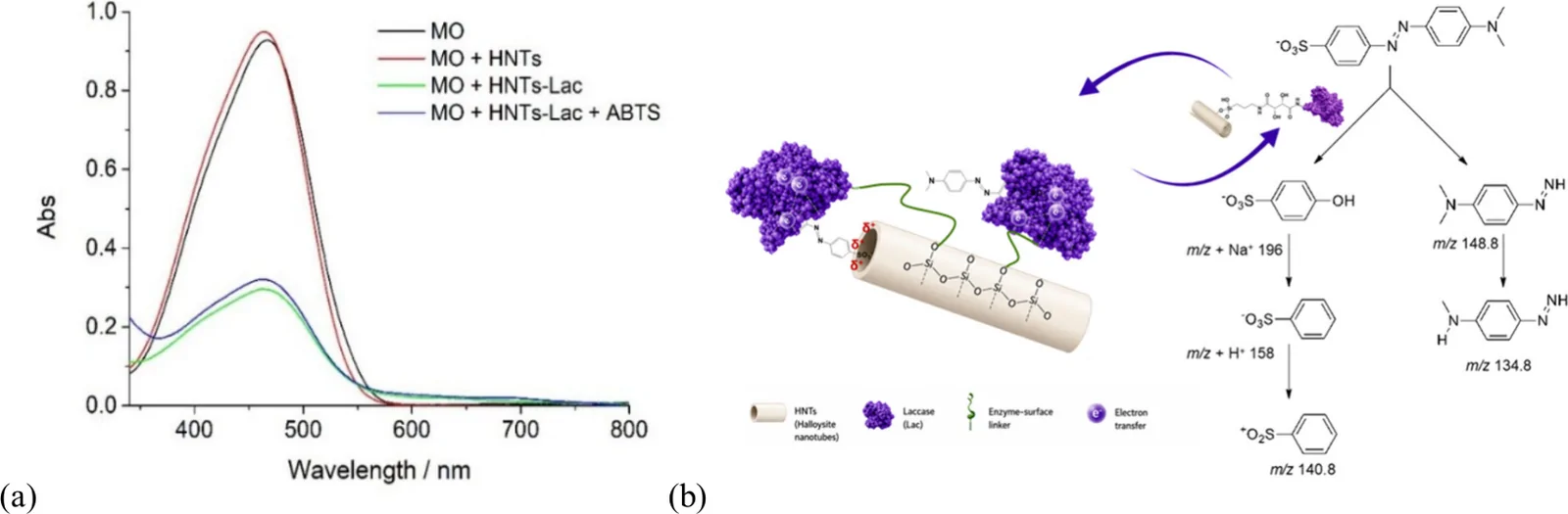
(a) UV–vis spectra of methyl orange (4 × 10^–5^ M) before (black and red lines) and after (green and blue lines) the reaction with HNTs-Lac, in the absence (green line) and presence (blue line) of ABTS; (b) Possible degradation pathway of MO in the presence of HNTs-Lac nanomaterial.

In laccase-based catalytic systems, the oxidation efficiency toward reducing substrates is strongly influenced by the structural organization and accessibility of the CuT1 catalytic site.^50^ Therefore, it is plausible that enzyme-surface interactions and interfacial confinement occurring after conjugation onto HNTs induce subtle structural and organizational rearrangements around the catalytic domain, ultimately affecting the catalytic response of the immobilized enzyme. These interfacial effects may contribute to the unusual mediator-free oxidation capability observed for the HNTs-Lac biomaterial. To further prove this hypothesis, the degradation of MO under identical conditions, specifically without the use of the ABTS mediator, by free laccase was investigated. As it is possible to observe from Figure S3, in this case unsatisfactory degradation was achieved, indicating the need to add ABTS as a redox mediator.

To understand the degradation mechanism of MO by HNTs-Lac biomaterial, ESI-MS analysis on the supernatant solution after degradation was performed (Figure S4). Based on the detected *m*/*z* values, several degradation intermediates were identified, suggesting that the process initially involves the cleavage of the azo bond (−N=N−), consistently with degradation pathways previously reported for laccase-mediated oxidation processes.^51^ The resulting asymmetric intermediates could subsequently undergo further oxidative transformations, as proposed in Figure 4b. These findings further demonstrate that the catalytic functionality of laccase is effectively preserved within the halloysite-based hybrid interface.

Figure 5a illustrates the reusability of the HNTs-Lac biomaterial in the degradation of MO (1 × 10^–4^ M). After each catalytic cycle, the biomaterial was recovered by centrifugation, thoroughly rinsed several times with water and methanol to eliminate residual compounds from the preceding reaction, and subsequently dried under vacuum before reuse. The recovered HNTs-Lac biomaterial was again added to a fresh MO solution, and its catalytic efficiency was evaluated again. Remarkably, the catalytic performance remained almost unchanged over at least six consecutive cycles, demonstrating the high operational stability and recyclability of the hybrid catalytic system. Finally, to prove the feasibility of the developed biomaterial for possible scale-up of the proposed methodology, a continuous flow technology, capable of preserving the effectiveness of the heterogeneous catalytic system and simplifying the recovery and reuse of the HNTs-Lac biomaterial, was adopted (Figure 5A). In particular, a glass column packed with a mixture of HNTs-Lac biomaterial and quartz sand (1:100 w/w) to ensure an elevated flow rate was linked to a solution reservoir and, through suitable tubing and valves, connected to a peristaltic pump.^52^ The reaction mixture was continuously recirculated through the thermostated column at 30 °C using a flow rate of 1.5 mL min^–1^ for 60 min. Subsequent UV–vis analyses revealed approximately 73% MO degradation, in good agreement with the batch experiments, further supporting the robustness and applicability of the developed dynamic redox-active hybrid system under continuous operational conditions. It is noteworthy that no MO degradation occurs when the MO solution was cycled pumped on simple quartz sand or a mixture of quartz sand with 1% of HNTs (Figure S5).

**Figure 5:**
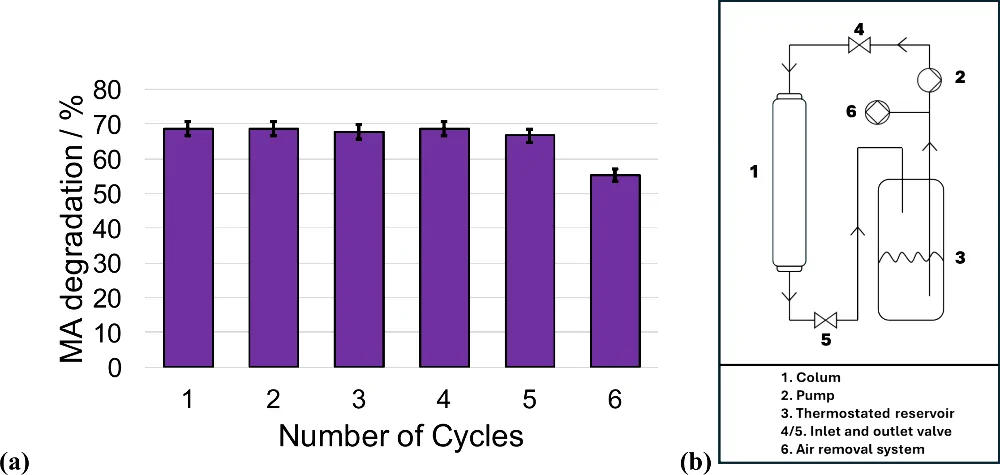
(a) Histogram showing the degradation efficiency of methyl orange (MO) at each reuse cycle of HNTs-Lac; data are mean values ± SD of three independent assays, (b) Schematic representation of the continuous-flow system employed for the degradation experiments.

Finally, Table 2 compares the catalytic performance of the HNTs-Lac biomaterial to that of other laccase-based materials reported for the degradation of organic dyes. As can be observed, the synthesized biomaterial exhibits promising activity for potential future applications, showing a degradation efficiency comparable to that of previously described systems, while notably operating without the addition of any mediator.

**Table 2:** Degradation of Organic Dyes by Various Laccase-Based Materials

**Immobilization material**	**Immobilization method**	**Degraded dye**	**Removal conditions**	**Decolorization efficiency (%)**	**References**
PCL/PEI	Cross-linking	OII MG	pH 5.0, 50 °C, 8 h	38.0–67.0	^53^
Lac@Cu-ZIF-90	Encapsulated	Reactive deep green	pH 4.0, 70 °C, 4 h	68.3–85.7	^54^
Chitosan-magnetic NP	Coated	Methyl Orange	pH 5.5, 40 °C, 1 h	60.0	^55^
M-HNTs-CTA-Lac	Coated	Direct Red 80	pH 4.2, 60 °C, 1 h	33.0	^56^
Fe_3_O_4_@C–Cu^2+^	Adsorption	few dyes	pH 4.5, 25 °C, 2 h	61.0	^57^
Sepharose-linked antibody	Coated	phenol red	pH 4.0, 50 °C, 2 h	44.0	^58^
Polymethacrylate/carbon nanotubes	Covalent coupling	OII	pH 5.0, 25 °C, 24 h	74.0	^59^
PEDOT–PPy-COOH/Pt	Covalent bonding	Procyon Red, R. Green 5, R. Brown 10, R. Green 19	pH 4.0, 75 °C, 10 min	76.0–94.0	^60^
Fe_3_O_4_-MWCNTs@SiO_2_	Cross-linking	R. Black 5	pH 3.0, 60 °C, 3.5 h	66.0	^61^
HNTs-Lac	Covalent bonding	Methyl Orange	pH 4.5, 30 °C, 1 h	68.5	This work

## 3 Conclusions

Two structurally distinct enzyme-halloysite hybrid systems were successfully developed through selective covalent conjugation onto the external surface of halloysite nanotubes, generating functional biointerfaces with preserved catalytic activity and high operational stability. Collagenase G (ColG), selected as a model proteolytic enzyme of biomedical relevance, was conjugated to amino-modified HNTs through EDC-mediated amide coupling. Conversely, laccase (Lac), chosen as a model redox biocatalyst for environmental applications, was interfaced with tartaric acid-modified halloysite (HNTs-COOH), a platform designed to improve enzyme accessibility and interfacial organization on the clay surface. The biomaterials were extensively characterized to assess the enzyme loading by thermogravimetric analysis and to confirm the successful modification by FT-IR spectroscopy and morphological investigation by SEM and HAADF/STEM investigation. Furthermore, DLS and z-potential measurements showed the presence of both enzymes on the external surface of the clay.

Enzymatic assays, using a fluorogenic reagent, in the case of the HNTs-ColG nanomaterial and ABTS for the HNTs-Lac one, demonstrated that both enzymes retained their catalytic properties after immobilization on halloysite, suggesting that the groups involved in the covalent linkage are not those responsible for enzymatic activity.

As a proof of concept, the HNTs-Lac nanomaterial was used as a catalyst for methyl orange degradation. Notably, the HNTs-Lac biomaterial exhibited unusual mediator-free catalytic behavior toward methyl orange degradation, achieving decolorization efficiencies comparable to those obtained in the presence of ABTS. These findings suggest that halloysite nanotubes do not merely behave as a passive support but rather as adaptive inorganic-biological interfaces capable of dynamically influencing enzyme function through interfacial effects, in particular, via the covalent-induced conformational modulation of the enzyme after its grafting onto the external HNTs surface. The developed biomaterial also exhibited excellent operational stability under both batch and continuous flow conditions, maintaining catalytic efficiency over multiple cycles and highlighting its potential applicability in sustainable wastewater remediation technologies.

Overall, this work demonstrates that halloysite nanotubes can serve not only as robust inorganic scaffolds for enzyme conjugation but, as a consequence of the covalent linkage of the enzymes, also as adaptive interfacial platforms capable of modulating the structural and catalytic behavior of immobilized enzymes. Beyond environmental applications, the HNTs-ColG system may also represent a promising platform for future biomedical strategies involving extracellular matrix modulation and advanced therapeutic nanomaterials.

Given the nonbiodegradability of halloysite, the developed systems hold significant promise for future applications in oral, topical, or localized administration. For instance, in the treatment of solid tumors, the administration of the HNTs-ColG system could facilitate the degradation of the extracellular matrix while simultaneously preventing premature enzyme inactivation. Furthermore, the empty lumen of the clay can be exploited for loading chemotherapeutic agents, thereby yielding a multifunctional nanomaterial with an enhanced biological performance.

## 4 Experimental Section

### 4.1 Synthesis of HNTs-Lac Biomaterial

In a 25 mL round-bottomed flask, 100 mg of HNTs-COOH and 50 mg of EDC were dispersed in 10 mL of anhydrous DMF and stirred for 10 min at room temperature. After this time, to the obtained dispersion, a laccase solution (10 mg, 2 mL, DMF) was added. The reaction mixture was maintained under stirring at room temperature for 48 h. Subsequently, the dispersion was centrifuged, and the recovered solid was repeatedly washed with water, to remove any Laccase interacting supramolecularly with HNTs, and then dried under a vacuum.

### 4.2 Synthesis of HNTs-ColG Biomaterial

In a 25 mL rounded bottom flask, 20 mg of ColG (1.5 × 10^–4^ mmol) and 1 mg of EDC (5.2 × 10^–3^ mmol) were dissolved in 15 mL of anhydrous DMF and stirred for 10 min at room temperature. After this time, to the obtained solution 100 mg of HNTs-NH_2_ were added. The reaction system was kept under continuous stirring at room temperature for 48 h. After completion of the process, the dispersion was centrifuged, and the obtained solid was repeatedly rinsed with water, to remove any ColG interacting supramolecularly with HNTs, and then dried under vacuum.

### 4.3 Activity Assay of Lac and HNTs-Lac Biomaterial

Laccase activity was determined by the ABTS oxidation method. The assay mixture containing ABTS (0.1 mM), sodium acetate buffer (0.1 mM; pH = 4.5), and an appropriate amount of enzyme (C = 5 × 10^–3^ mg mL^–1^) was stirred at 30 °C, and the absorbance of the solution was measured by UV–vis at set time for 60 min.

Immobilized laccase activity on HNTs was determined by dispersing 1 mg of HNTs-Laccase (C_Laccase_ = 5 × 10^–3^ mg mL^–1^) in 3 mL of a mixture of sodium acetate (0.1 mM; pH = 4.5) and ABTS (0.1 mM). The reaction mixture was stirred for 30 min at 30 °C, centrifuged, and the supernatant was measured by UV–vis. The same experiment was performed using pristine HNTs as control.

In addition, HNTs-Laccase biomaterial activity was also determined by modified ABTS assay, which involved dispersing 4 mg of HNTs-Lac biomaterial in 1 mL of a mixture of citrate buffer (100 mM, pH = 3.0) and ABTS (1.0 mg mL^–1^). The dispersion was stirred for 15 min at 30 °C and centrifuged, and the supernatant was measured by UV–vis. The same procedure was repeated using pristine HNTs as control.

### 4.4 Absorption of the ABTS Radical onto HNTs

To evaluate the adsorption of the ABTS radical by HNTs, an ABTS solution (0.1 mM) was stirred under light exposure for 24 h to generate characteristic blue-green radical species. Subsequently, 5 mg of HNTs was added to the resulting solution, and the suspension was stirred for 5 min. After this period, the absorbance of both the dispersion and the reference solution was measured by UV–vis spectroscopy.

### 4.5 Activity Assay of ColG and HNTs-ColG Biomaterial

100 μL of a standard collagen solution were sequentially combined with 20 μL of HNTs-ColG dispersion (1 mg mL^–1^) or ColG solution (0.1 mg mL^–1)^ in TRIS buffer pH 7.5, 100 μL of a buffer mixture containing 125 mM sodium borate (pH 7.5) and 5 mM CaCl_2_, and 30 μL of deionized water. The mixture was then incubated at 37 °C for 60 min. After this time, 250 μL of the enzymatic reaction mixture were combined with 250 μL of 0.75 mM 3,4-DHPAA in water, 250 μL of 125 mM sodium borate buffer (pH 8.0), and 250 μL of 1.25 mM NaIO_4_ in water. The reaction was immediately incubated at 37 °C for 10 min.

### 4.6 Methyl Orange Degradation Using HNTs-Lac

In a glass vial, 4 mg of HNTs-Lac were dispersed in 800 μL of 0.1 mM acetate buffer (pH 4.5). Pristine HNTs were used as the control sample. The dispersion was equilibrated at 30 °C for 10 min, followed by the addition of 200 μL of a methyl orange (MO) solution (C = 2 × 10^–4^ M), resulting in a final MO concentration of 4 × 10^–5^ M. The mixture was subsequently incubated at 30 °C for 1 h. Following incubation, the dispersion was centrifuged, and the resulting supernatant was recovered for UV–vis spectroscopic analysis.

### 4.7 Reusability of HNTs-Lac Biomaterial

A total of 10 mg of HNTs-Lac were placed in a 30 mL Corex tube and dispersed in 2 mL of acetate buffer solution (0.1 mM, pH 4.5). Then, 0.5 mL of a methyl orange solution (2 × 10^–4^ M) was added, and the mixture was kept under constant stirring at 30 °C for 1 h. After this time, the dispersion was centrifuged, the supernatant was collected, and the UV–vis spectrum was recorded. The solid residue was thoroughly washed by centrifugation with water and methanol and then dried under vacuum at 25 °C. For subsequent reuse cycles, the biomaterial was accurately weighed each time, and the volumes of buffer and methyl orange solution were adjusted proportionally.

### 4.8 Degradation of Methyl Orange under Flow Conditions

A glass column (Supelco Omnifit Column Kit) with an internal diameter of 10 mm and a length of 100 mm was filled with sand (50–70 mesh) containing 1 wt % of the HNTs-Lac catalyst. Initially, the column was conditioned with acetate buffer (0.1 mM, pH 4.5) delivered by a peristaltic pump (Shimadzu LC-10AD VP). To assess the catalytic efficiency of the system, a mixed solution of methyl orange (MO, 4 × 10^–4^ M) was placed in a round-bottom flask serving as the reservoir. The setup was then linked to the peristaltic pump by means of suitable tubing and valves. The reaction mixture was cyclically circulated (flow rate of 1.5 mL min^–1^) through the column containing the HNTs-Lac biomaterial at 30 °C for 60 min. The reaction was monitored using UV-*vis* spectroscopy. For reactor cleaning, water was cyclically circulated through the column. Same experiment was performed using only sand or sand mixed with 1% of pristine HNTs.

## Supplementary Material



## Notes

Instruments. Calculation of the enzyme loading. Figures S1 Emission spectra of the fluorescent NGPs-3,4-DHPAA compound (λ_ex_ = 365 nm) after enzymatic digestion of collagen-I by means of ColG; Figure S2: UV-*vis* spectra of ABTS 0.05 mM in the presence of HNTs (1 mg mL^–1^) at different time (0 – 60 min), Figure S3: UV–vis spectra of methyl orange (4 × 10–5 M) in the presence and absence of free laccase, Figure S4: ESI-MS spectrum (negative ion mode) of MO supernatant solution after degradation in the presence of HNTs-Lac nanomaterial, Figure S5: Degradation of MO under flow conditions in the presence of only quartz sand, mixture of quartz sand and HNTs (1%) and quartz sand and HNTs-Lac (1%) (PDF)
