# Supplementary material for: Halloysite Nanotubes as Dynamic Interfaces for Functional Enzyme Biomaterials
Source: ACS Omega. 2026 Aug 17;11(34):51690–700. doi: 10.1021/acsomega.6c06020 (PMC13625072; doi:10.1021/acsomega.6c06020)
Supplement: Supplementary file 1 [file ao6c06020_si_001.pdf]

Supporting information

Halloysite Nanotubes as Dynamic Interfaces for Functional Enzyme Biomaterials

Giuseppe Cinà,[1] *Marina Massaro,*[2] *Federica Leone,*[1] *Claudia Sciacca,*[1] *Nunzio Cardullo,*[1] *Vera Muccilli,*[1] *Angelo Nicosia,*[1] *Placido G. Mineo,*[1] *Rita Sánchez-Espejo,*[3] *César Viseras,*[3,4] *Serena Riela*[*,1]

[1]Dipartimento di Scienze Chimiche (DSC), Università di Catania, Viale Andrea Doria 6, 95125, Catania, Italy.

[2]Dipartimento di Scienze e Tecnologie Biologiche, Chimiche e Farmaceutiche (STEBICEF), Università degli Studi di Palermo, Viale delle Scienze, Parco d'Orleans II, Ed. 17, 90128 Palermo, Italy.

[3]Department of Pharmacy and Pharmaceutical Technology, School of Pharmacy, Campus of Cartuja s/n, University of Granada, 18071, Granada, Spain.

[4]Andalusian Institute of Earth Sciences, CSIC-UGR, 18100 Armilla, Granada, Spain

Table of contents

1. Instruments……………………………………………………………………………………….S3

2. Calculation of the enzymes loading from thermogravimetric data…………………………….…S5

3. Emission spectrum of the fluorescent NGPs-3,4-DHPAA in the presence of ColG……………….S6

4. UV-*vis* spectra of ABTS in the presence of pristine HNTs………………………………………...S7

5. UV-*vis* spectra of methyl orange in the presence and absence of free laccase……………………S8

6. ESI-MS spectrum of MO supernatant solution after degradation…………………………………S9

7. Degradation of MO under flow conditions in the presence of only quartz sand………………….S10

8. References…………………………………………………………………………………….S11

Instrumets

All reagents used for biomaterial synthesis were purchased from Merck and used without further purification. Laccase used in this study was purchased from Sigma-Aldrich and was Laccase from *Trametes versicolor*. The ultrapure recombinant collagenase ColG (145 kDa) was provided by Abiel s. r. l. (Palermo, Italy); unlike other commercial collagenases, of extractive origin which contain various contaminants with proteolytic activity, the collagenases produced by ABIEL srl are recombinant enzymes of class I of *Clostridium hystoliticum*, which have been optimized to be produced in *Escherichia coli*, in the strain BL21 Ai, with a purity > 99% and totally free of contaminants with enzymatic activity.[1] Synthesis of HNTs-NH_2 and HNTs- COOH are reported elsewhere.[2]

FT-IR spectra (KBr) were acquired by means of an Agilent Technologies Cary 630 FT-IR spectrometer.

Thermogravimetric analyses were performed with a Perkin-Elmer TGA 7 equipped with a TAC 7/DX (Perkin Elmer, Waltham, MA, USA), in the range from 50 to 800 °C (thermal ramp of 10 °C min^{-1}) and N_2 atmosphere (60 mL min^{-1}).

The size analysis, ζ-potential and polydispersity index of the samples were determined using a Malvern Zetasizer Nano ZS instrument, fitted with a 632 nm laser at a fixed scattering angle of 173°.

Morphologies were imaged by means of a FEI Titan G2 60-300 ultra-high resolution transmission electron microscope (FEI, Lausanne, Switzerland) coupled with analytical electron microscopy (AEM) performed with an energy dispersive X-ray spectroscopy (XEDS) detector. AEM spectra were saved in scanning transmission electron microscopy (STEM) mode with a high angle annular dark field (HAADF) detector. Elemental maps were also collected using X-rays.

Scanning electron microscopy (SEM) images were acquired with a GEMINI (FESEM) CARL Zeiss coupled to an EDX microanalyser (Oxford Instruments). Samples were dried at 40 °C for a minimum of 48 h. Then, powdery samples were mounted over standard aluminum stubs and coated with carbon.

Fluorescence spectra were acquired by using a JASCO FP-8300 spectrofluorometer (JASCO, Cremella (LC), Italy). For solution measurements, the excitation and emission slit widths were set to 5 nm and 2.5 nm, respectively, and spectra were recorded over the wavelength range of 390-700 nm.

Diffuse reflectance UV-*vis* spectra were acquired using an Avantes spectrophotometric system composed of an AvaLight DH-S-BAL light source, an AvaSpec-ULS2048CL-EVO detector, and a FCR-7UVIR200-2-BX fiber-optic probe equipped with a collimating lens. Measurements were performed in diffuse reflectance mode under ambient conditions, and the spectra were collected over the UV-*vis* spectral range.

Electrospray ionization mass spectra (ESI-MS) were acquired using a Thermo Finnigan LCQ DECA mass spectrometer, operating in negative ion mode, with water as the solvent.UV-vis measurements were acquired with a Beckmann DU 650 spectrometer.

Calculation of the enzyme loading from thermogravimetric data [3-4].

The amount of enzymes grafted onto HNTs external surface was calculated by using the rule of mixtures for the degraded matters at 800 °C (MD_{800}).

MD_{800} values are calculated as follows:

$$MD_{800} = 100 - (MR_{800} + ML_{150}) \qquad \text{Eq. 1}$$

where MR_{800} is the residual matter at 800 °C, while ML_{150} is the mass loss between 25 and 150 °C. ML_{150} represents the water content of the investigated material.

As far is regarded HNTs-ColG and HNTs-Lac nanomaterials, the $MD_{800\text{-HNTs-Enzymes}}$ can be expressed as

$$MD_{800-HNTs-Enzymes} = \frac{C_{HNTs-NH_2} \times MD_{800-HNTs-NH_2}}{100} \qquad \text{Eq. 2}$$

where $C_{HNTs-NH_2}$ is the weight percent of HNTs-NH_2, while $MD_{800-HNTs-NH_2}$ is the degraded matters at 800 °C for the corresponding pristine components.

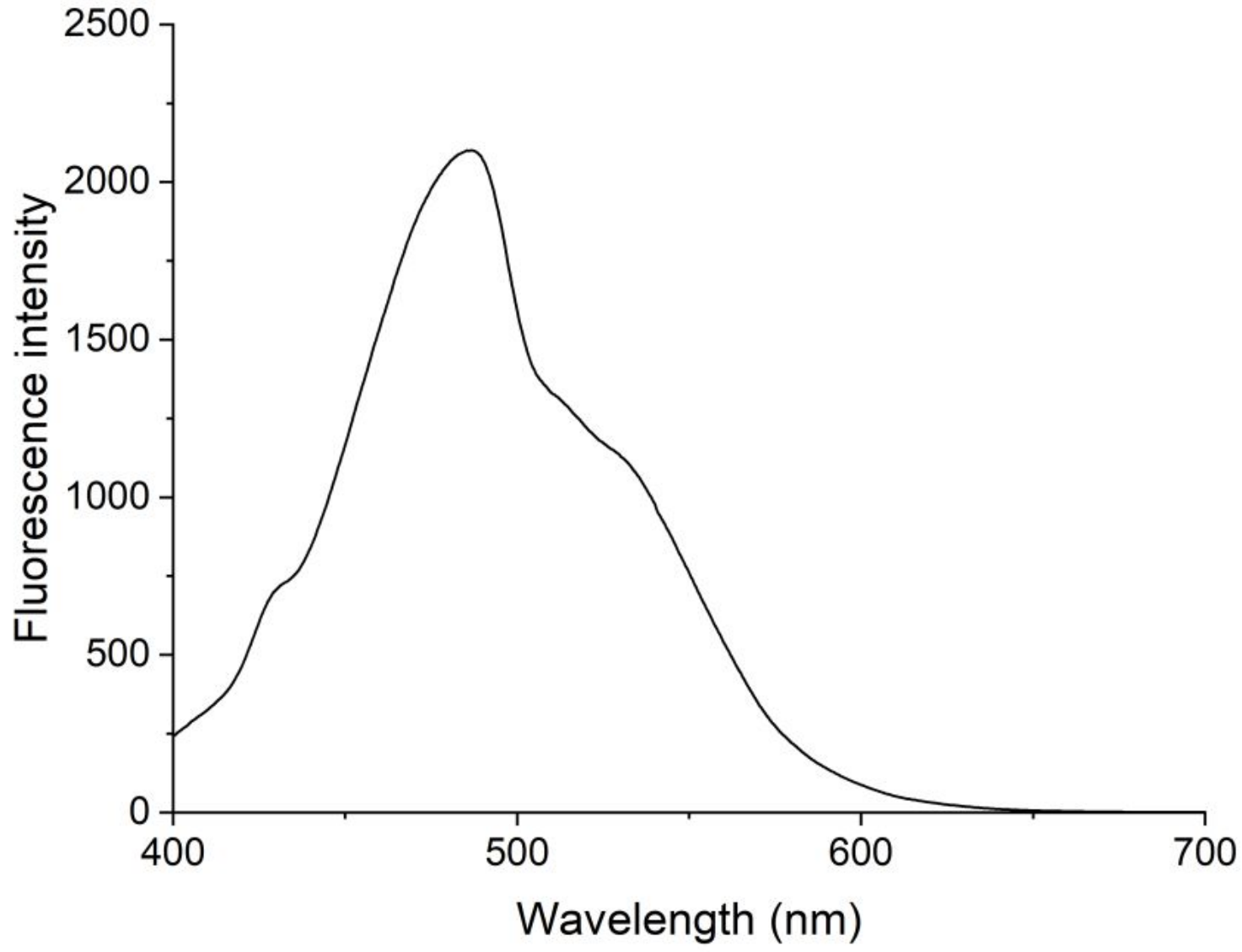

Figure S1. Emission spectra of the fluorescent NGPs-3,4-DHPAA compound (λ_{ex} = 365 nm) after enzymatic digestion of collagen-I by means of ColG.

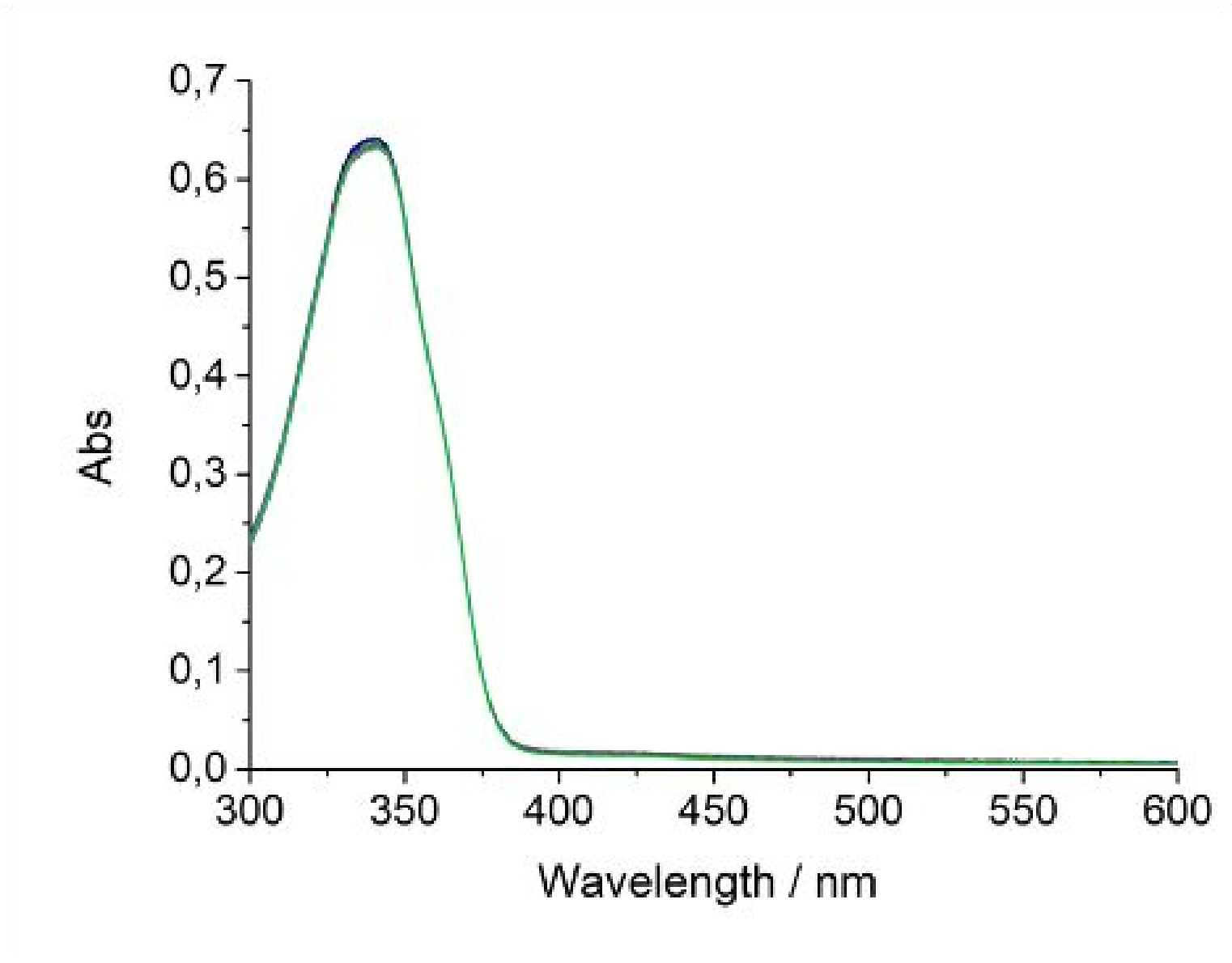

Figure S2. UV-*vis* spectra of ABTS 0.05 mM in the presence of HNTs (1 mg mL^{-1}) at different time (0 – 60 min).

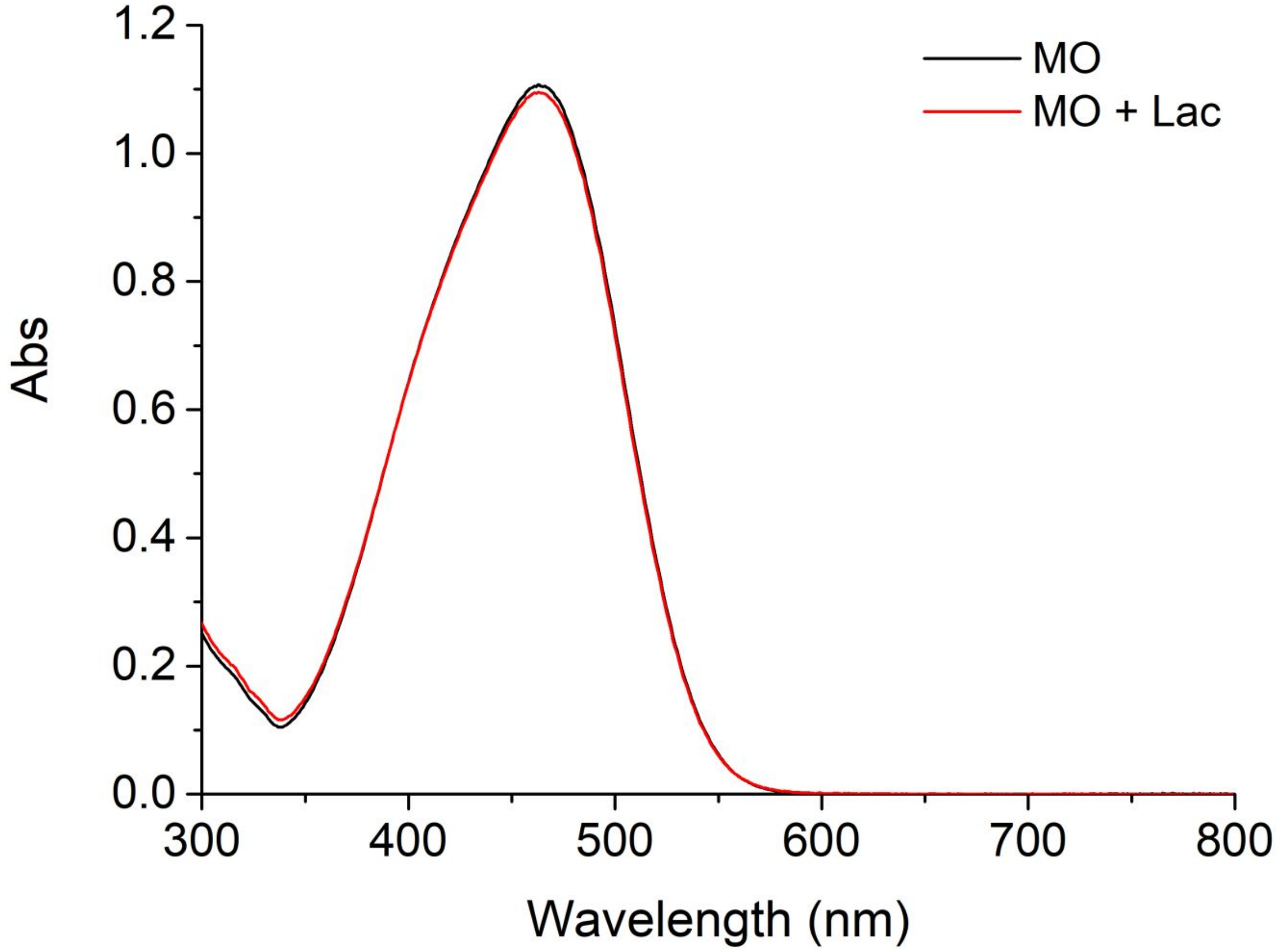

Figure S3. UV-*vis* spectra of methyl orange (4 × 10^{-5} M) in the presence and absence of free laccase.

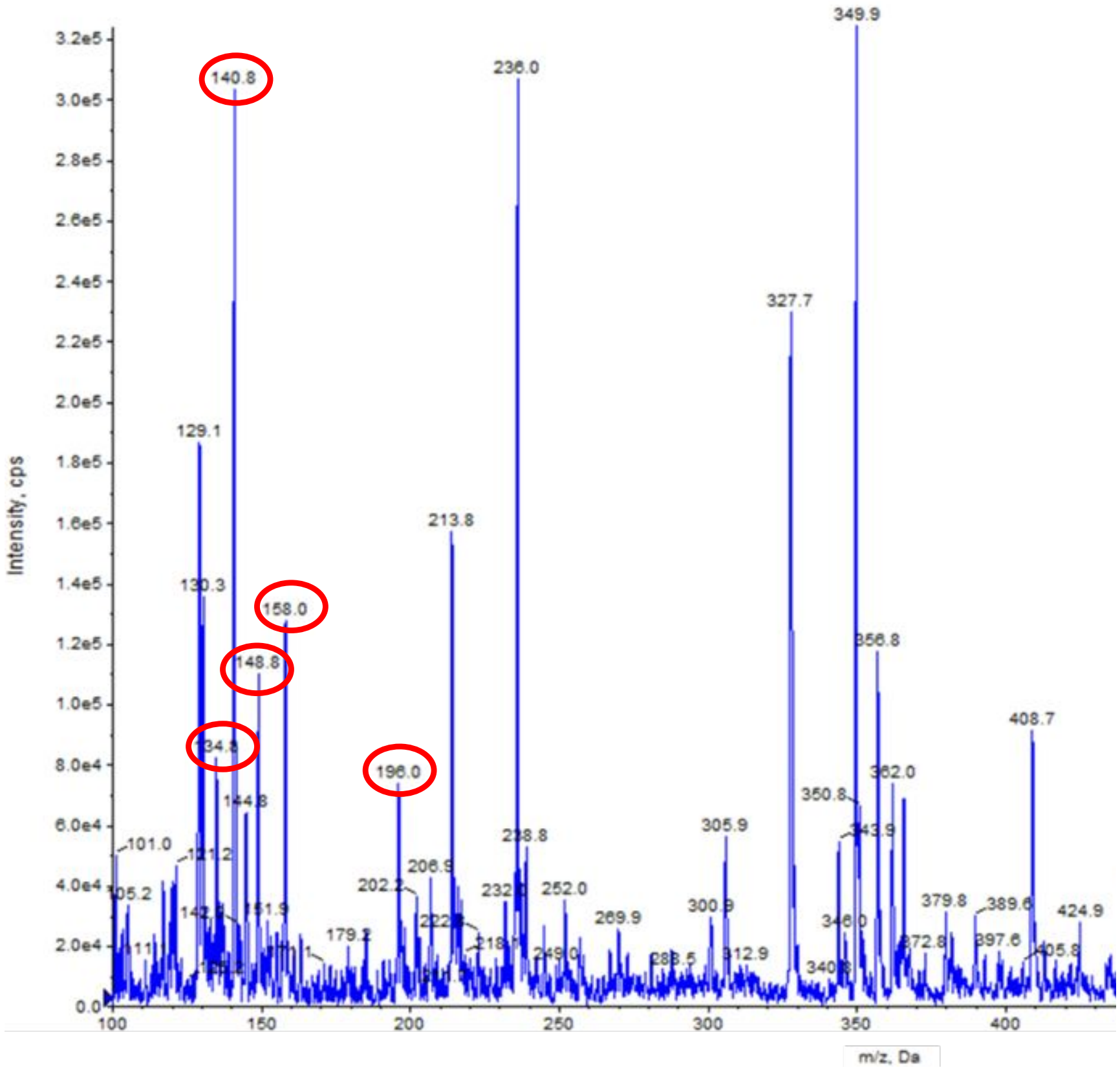

Figure S4. ESI-MS spectrum (negative ion mode) of MO supernatant solution after degradation in the presence of HNTs-Lac nanomaterial.

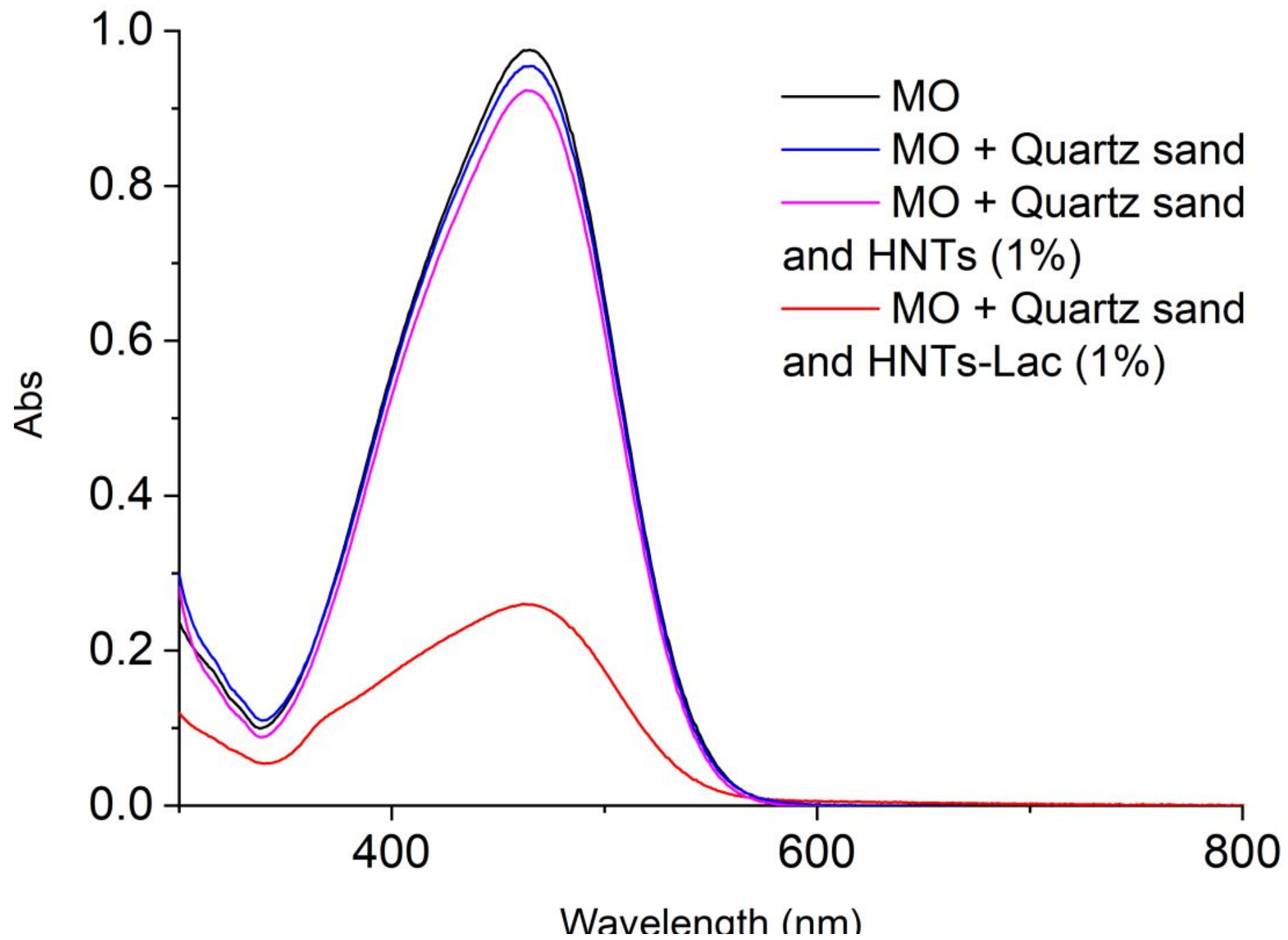

Figure S5. Degradation of MO under flow conditions in the presence of only quartz sand, mixture of quartz sand and HNTs (1%) and quartz sand and HNTs-Lac (1%).

References

1. Volpe, L.; Salamone, M.; Giardina, A.; Ghersi, G., Optimization of a Biotechnological Process for Production and Purification of Two Recombinant Proteins: Col G and Col H. *Chemical Engineering Transactions* **2016,** *49*, 61-66.
2. Cinà, G.; Massaro, M.; Cavallaro, G.; Lazzara, G.; Sánchez-Espejo, R.; Viseras Iborra, C.; D'Abrosca, B.; Fiorentino, A.; Messina, G. M. L.; Riela, S., Development of alginate film filled with halloysite-carbon dots for active food packaging. *International Journal of Biological Macromolecules* **2024,** *277*, 134375.
3. Lisuzzo, L.; Cavallaro, G.; Milioto, S.; Lazzara, G., Halloysite Nanotubes Coated by Chitosan for the Controlled Release of Khellin. *Polymers* **2020,** *12* (8), 1766.
4. Lisuzzo, L.; Cavallaro, G.; Pasbakhsh, P.; Milioto, S.; Lazzara, G., Why does vacuum drive to the loading of halloysite nanotubes? The key role of water confinement. *J. Colloid Interface Sci.* **2019,** *547*, 361-369.
